# Single Nucleotide Polymorphisms’ Causal Structure Robustness within Coronary Artery Disease Patients

**DOI:** 10.3390/biology12050709

**Published:** 2023-05-12

**Authors:** Maria Ganopoulou, Theodoros Moysiadis, Anastasios Gounaris, Nikolaos Mittas, Fani Chatzopoulou, Dimitrios Chatzidimitriou, Georgios Sianos, Ioannis S. Vizirianakis, Lefteris Angelis

**Affiliations:** 1 School of Informatics, Aristotle University of Thessaloniki, 54124 Thessaloniki, Greece; 2 Department of Computer Science, School of Sciences and Engineering, University of Nicosia, Nicosia 2417, Cyprus; 3 Department of Chemistry, International Hellenic University, 65404 Kavala, Greece; 4 Laboratory of Microbiology, School of Medicine, Aristotle University of Thessaloniki, 54124 Thessaloniki, Greece; 5 Labnet Laboratories, 54638 Thessaloniki, Greece; 6 First Department of Cardiology, AHEPA University General Hospital of Thessaloniki, 54124 Thessaloniki, Greece; 7 Laboratory of Pharmacology, School of Pharmacy, Aristotle University of Thessaloniki, 54124 Thessaloniki, Greece; 8 Department of Health Sciences, School of Life and Health Sciences, University of Nicosia, Nicosia 2417, Cyprus

**Keywords:** causal models, coronary artery disease, Markov Blanket, Syntax Score

## Abstract

**Simple Summary:**

Coronary artery disease is one of the major cardiovascular diseases associated with multiple inherited and environmental risk factors, and constitutes one of the leading causes of mortality globally. Due to the exceptionally high mortality rates and incidence of coronary artery disease, there exists a pivotal need for the development of methodological algorithms for the prediction of its complexity and severity. This work aimed to assess the robustness of the relationships of previously identified, important genetic risk factors affecting the Syntax Score, an index that evaluates the complexity of the disease. The analysis shows that these relationships were quite robust under mild interventions, whereas in the case of stronger interventions, they were more sensitive. In addition, it validated a set of risk factors capable to predict future cardiovascular events in individuals and specific populations. Thus, this work may contribute to the advancement of the data analytics and algorithm development relevant to coronary artery disease patient handling, therapeutic decisions, and predictions of severity and complexity of the disease.

**Abstract:**

An ever-growing amount of accumulated data has materialized in several scientific fields, due to recent technological progress. New challenges emerge in exploiting these data and utilizing the valuable available information. Causal models are a powerful tool that can be employed towards this aim, by unveiling the structure of causal relationships between different variables. The causal structure may avail experts to better understand relationships, or even uncover new knowledge. Based on 963 patients with coronary artery disease, the robustness of the causal structure of single nucleotide polymorphisms was assessed, taking into account the value of the Syntax Score, an index that evaluates the complexity of the disease. The causal structure was investigated, both locally and globally, under different levels of intervention, reflected in the number of patients that were randomly excluded from the original datasets corresponding to two categories of the Syntax Score, zero and positive. It is shown that the causal structure of single nucleotide polymorphisms was more robust under milder interventions, whereas in the case of stronger interventions, the impact increased. The local causal structure around the Syntax Score was studied in the case of a positive Syntax Score, and it was found to be resilient, even when the intervention was strong. Consequently, employing causal models in this context may increase the understanding of the biological aspects of coronary artery disease.

## 1. Introduction

Advances in technology in recent years are leading to a constant increase in the accumulation of data, and pose a challenge in exploiting these data in order to extract valuable information and uncover new knowledge. Causal models are a powerful tool that can be used within different setups towards this direction. Their aim is to unveil the structure of causal relationships between different variables. A significant advantage in causal relationships between variables is the existence of direction, which determines the cause and the effect in each relation. This is of particular importance in several scientific fields, including biology. The causal structure in a dataset may aid experts in a field to better understand relations, or even extract new knowledge.

Coronary artery disease (CAD) is one of the major cardiovascular diseases associated with multiple inherited and environmental risk factors, and it remains one of the leading causes of mortality globally. Due to the exceptionally high mortality rates of CAD and its incidence in both developed and developing countries worldwide, there is a pivotal need for the development of risk-stratification machine learning algorithms for the prediction of CAD complexity and severity [1,2]. Genes whose dysfunction has been implicated in the molecular pathophysiology and genetics of CAD include those involved in the regulation of blood pressure (e.g., CYP17A1), nitric oxide signaling (e.g., NOS3), the initiation of plaque formation (e.g., APOB, PCSK9, LRP1) and progression (e.g., LOX, COL4A1/2, ANRIL), cellular proliferation (e.g., CDKN2A/B, SMARCA4, PDS5B), inflammation (e.g., IL5, CXCL12, PLG, TRIM22), angiogenesis (e.g., TGFB1, FGD6, VEGFA), and transcriptional regulation (e.g., BACH1, HDAC5, FGF5) [3,4].

In a previous study of our group [5], the dynamics of causal models were employed to investigate the structure of causal relationships between single nucleotide polymorphisms (SNPs) and the Syntax Score in patients with CAD from the GESS clinical trial (ClinicalTrials.gov Identifier: NCT03150680, [6]). The genetic analysis and information about the SNPs included in the GESS clinical trial have been CRIS-published elsewhere [4]. The Syntax Score is an index that evaluates the complexity of CAD. The causal structure of the SNPs was assessed when the Syntax Score was zero, and, separately, when it was positive. Then, the cause–effect relationships between the SNPs and the Syntax Score (for positive values only) were locally investigated. Directed Acyclic Graphs (DAGs) were employed, and the local causal structure around the Syntax Score was assessed. The segregation of the Syntax Score into these two categories (zero and positive) was based on the application of a two-part machine learning modeling approach that was previously proposed for the prediction of the Syntax Score in [7]. It was found [5] that the obtained causal relationships concerned SNPs that were related to important genes in cancer and cardiovascular functions. In the current study, the motivation was to assess the robustness of the causal structures obtained in [5]. To this end, a sensitivity analysis’ methodological approach is proposed herein, which assesses the impacts of different levels of interventions in these causal structures. The biological implications of this analysis concern the reliability of the biological interpretations emerging from the obtained causal structures in [5], and further support the biological conclusions therein.

Causal models have been used in the literature within different contexts, and by employing different methodological schemes. Li et al. urged scientists from different fields to exploit the potential of causal models in real-life applications [8]. Causal structure investigation, and DAGs, have been employed by authors of this work in several studies. For example, in [9], causal discovery was employed in sweet cherry multi-omics data, leading to understanding of cause–effect relationships that are important in the fruit softening and ripening process. In addition, a proteogenomic-based causal model analysis unveiled key interaction networks that are involved in salt priming in olive trees [10]. Causal models were employed as well in a multi-omics analysis, aiming to determine the molecular portrait of the famous PGI Naxos Island’s potatoes, that revealed key environment-derived molecular factors [11].

The local causal structure around a specific variable of interest, and in particular the Markov Blanket of a variable, is widely used in the literature. Piccininni et al. performed an extensive simulation study, and assessed the usage of the Markov Blanket for feature selection compared to other methodological approaches [12]. It was empirically demonstrated that a Markov Blanket-based model performed similarly to or better than all its competitors, and it was concluded that utilizing the Markov Blanket around a target variable may be an efficient strategy to identify the most important predictors in clinical risk predictive models. A causal graph-based modeling approach employed the concept of the Markov Blanket in [13], and found it to perform better compared to other models regarding lung cancer prediction. Ganopoulou, et al., assessed the effectiveness of the Markov Blanket as a feature selection tool to detect predictors that are causally related to the result of the percutaneous coronary interventions (PCI) in chronic total occlusions [14]. A customized predictive model of the PCI result, which included these predictors, was compared to other modeling approaches from the literature, and was found to perform equally well or better. Other studies that employed the Markov Blanket include, but are not limited to, [15,16,17,18,19].

The aim in this study was to perform a sensitivity analysis to assess the robustness of the causal structures between SNPs and the Syntax Score in patients with CAD. In particular, the analysis was based on genomic information related to 228 SNPs, and the value of the Syntax Score, for 963 patients with coronary artery disease. The sensitivity analysis involved three main pillars. (I) Within the first pillar, the robustness of the SNPs’ causal structure was assessed based on CAD patients that exhibited a zero Syntax Score (267 cases). Then, it was independently assessed based on CAD patients that exhibited a positive Syntax Score (597 cases). (II) In the second pillar, the robustness of the similarity between the SNPs’ causal structures corresponding to zero and positive Syntax Scores was assessed. (III) The third pillar of the analysis was focused on assessing the robustness of the SNPs’ local causal structure around the Syntax Score, reflected in its Markov Blanket. The assessment of the Markov Blanket was performed only in the case that the Syntax Score was positive.

The analysis has shown that the causal structure of the SNPs was influenced, as expected, to a higher extent when the interventions were stronger, and to a much lower degree under milder interventions. The Markov Blanket around the Syntax Score, in particular, has been shown to be resilient, even when stronger interventions were employed.

## 2. Materials and Methods

### 2.1. Causality

Causal discovery justifies the causal nature of a relationship between two variables based on its persistence, thus going beyond traditional statistical association assessment [8]. Persistence is the main attribute of a causal association. Investigating for the existence of a causal association between two variables in a dataset involves all the remaining variables of this dataset, and considers all circumstances [20]. More specifically, the causal nature of the association is expected to exist in all situations, and not be influenced by the values of the remaining variables. Consequently, causal relationships are expected to be less volatile compared to standard statistical associations. An additional advantage of causal model development is the existence of a direction in the causal relationships between variables, which characterizes the cause and the effect variable in each relation [8]. This is of particular importance in several scientific fields, including biology, and contrasts with the typically used correlation indices, which are mostly bidirectional.

### 2.2. PC Algorithm and the Markov Blanket

The constrained-based PC algorithm [21,22] is a method that is commonly used to learn the structure of a causal Bayesian network. The algorithm is named after its inventors, Peter Spirtes and Clark Glymour [21]. Specifically, for every pair of variables (X,Y) in a dataset of interest, the PC algorithm assesses their conditional independence given all the remaining variables. Practically, the algorithm assesses their association conditioning on all subsets of all variables other than X and Y, in order to determine whether the association between X and Y is persistent [22]. In case X and Y are conditionally independent, the algorithm indicates a nonexistent causal relationship between X and Y; thus, no edge should be drawn between X and Y in the corresponding graph. On the other hand, the relationship is considered to be causal when the association exists given each of the conditioning sets. It is typically assumed that causal sufficiency holds [8]. This condition implies that for each pair of measured variables, all their common direct causes are measured as well. That is to say, there are no hidden, unmeasured confounders for any pair of variables.

After the causal relationship between variables has been determined, a network with a structure reflecting the results of the tests of independence is developed. This network is represented by a Markov equivalence class of the directed acyclic graph. All DAGs that belong in a specific equivalence class describe the same conditional independence relationships, since they are structured with the same skeleton (adjacencies) and the same v-structures. Assume D is a DAG. The skeleton of D is the undirected graph that is formed by removing directions of all the edges in the DAG. A v-structure in D is an ordered triplet of nodes (x,y,z), such that D contains the directions of x→y and y←z; additionally, the nodes x,z are not connected with an edge in D. Some edges, however, may exhibit an undetermined direction (so-called bidirected/undirected edges). This means that they have the opposite direction from one DAG in the equivalence class to another DAG in the same equivalence class. There is an edge (directed or bidirected) between the variables/nodes x and y, if, and only if, the variables are conditionally dependent given S, for all possible subsets S of the remaining variables/nodes [23].

By definition, the Markov Blanket of a variable T is the minimal variable subset conditioned on which all other variables are probabilistically independent of T. The Markov Blanket of T consists of the variables representing its parents, children, and other parents of its children in the graph (spouses). It is expected to contain the most informative variables for the variable T.

### 2.3. Data Description

The sensitivity analysis was based on 963 patients with coronary artery disease from the GESS clinical trial (ClinicalTrials.gov Identifier: NCT03150680) [6]. For these patients, there was information available regarding 228 SNPs, and the Syntax Score value.

### 2.4. Statistical Analysis

The dataset initially consisted of 963 cases/patients, and 229 variables (228 SNPs, and the Syntax Score). The data were then assessed for missing values, and cases with at least one missing value in any variable were excluded (99 in total), resulting in 864 cases in total. The statistical analysis addressed the three main pillars.

(I) The first pillar concerned the assessment of the robustness of the SNPs’ causal structure, taking into account the Syntax Score’s values. In particular, the causal structure of the SNPs was determined at first in the case that the Syntax Score was equal to zero (n = 267 cases). This structure is represented by SS0. Then, the causal structure of the SNPs was independently obtained in the case that the Syntax Score was higher than zero (n=597 cases). This structure is represented by SS1. In each case, the causal relationships among SNPs were determined with the constrained-based PC algorithm, using the R package “MXM” [24]. The skeleton of the causal network was developed with the function “pc.skel”, using the “comb.mm” method (to assess the conditional independence for every pair of variables, each of the two variables is treated as a response and the appropriate regression model is fitted. Next, two likelihood ratio tests are performed, and the two emerging *p*-values are combined in an overall *p*-value), and by setting alpha to 0.005. Next, both structures were assessed for robustness.

The assessment of the robustness was performed independently for the causal structures SS0 and SS1, by imposing different levels of intervention. The intervention was reflected in the number of patients, k, that were randomly excluded from the corresponding dataset (n=267,n=597, respectively). More specifically, in the case of SS0, k=1,2,3,5,10,20,30,50 patients who were randomly selected and excluded from the corresponding dataset (n=267). The numbers that k assumed were arbitrarily selected to provide a wide range of interventions, with higher numbers of k representing stronger interventions. The remaining n−k patients were used to re-determine the causal structure of the SNPs. The above procedure was repeated 100 times for each k. Then, these 100 SNPs’ causal structures were compared, on average, to SS0. A similar procedure was followed in the case of SS1. The reason to repeat the exclusion procedure 100 times for each k was to obtain more balanced information, averaged over the corresponding 100 SNPs’ re-determined/new causal structures.

Several metrics were considered to compare the initial causal structures (SS0 and SS1) to the mean behavior of the 100 new SNPs’ causal structures that were obtained from the corresponding datasets (n=267,n=597, respectively), after randomly excluding k=1,2,3,5,10,20,30,50 patients. All these metrics were applied, for each k, independently, in the case of SS0 and SS1. These metrics will be described below in the case of SS0 (similarly, they were computed in the case of SS1). At first, for each k, the mean number, over the 100 new SNPs’ causal structures of bidirected edges that were in common with SS0 was computed, along with the corresponding mean percentage. Similarly, the mean number of directed edges that were in common with SS0 and the corresponding mean percentage were computed. On top of that, the mean number of directed edges in SS0 that turned into bidirected edges in the 100 new SNPs’ causal structures was recorded, along with the corresponding mean percentage. Similarly, the mean number of directed edges in SS0 that changed direction in the 100 new SNPs’ causal structures was computed as well, along with the corresponding mean percentage. Next, the structural Hamming distance, as defined in [25], was used. This distance accounts for the number of operators required to make two partially oriented DAGs match, or more specifically, to add or delete an undirected edge, and add, remove, or reverse the orientation of an edge. Thus, for each k, the mean structural Hamming distance, over the 100 new SNPs’ causal structures, to SS0 was computed. A mean relative structural Hamming distance was computed as well, by dividing the actual measured mean structural Hamming distance by the maximum value that it could potentially receive in each case, i.e., if all relationships between nodes were altered (e.g., a directed relationship turned into a bidirected one).

The reasoning underlying these customized metrics was to assess the impact inflicted independently upon each of the initial causal structures, SS0 and SS1, when k patients were randomly excluded from each corresponding dataset (n=267,n=597, respectively), thus providing an assessment of their robustness.

(II) In the second pillar, the robustness of the similarity between SS0 and SS1 was assessed. The two causal structures were compared, at first using the structural Hamming distance. The number of common bidirected and directed relationships was recorded as well. Then, the same number of k=1,2,3,5,10,20,30,50 patients were randomly selected and excluded from each dataset (n=267,n=597, respectively), and the corresponding two SNPs’ causal structures were re-determined, based on the remaining n−k patients in each case, and compared to each other. The above procedure was repeated 100 times for each k.

Then, for each k, the mean number of common bidirected and directed relationships, over the 100 pairwise comparisons, between the re-determined SNPs’ causal structures corresponding to SS0 and SS1, was recorded. On top of that, the mean ratio of these common numbers of bidirected and directed relationships to the corresponding numbers of common bidirected and directed relationships, respectively, between SS0 and SS1, was computed. Additionally, for each k, the mean structural Hamming distance over the 100 pairwise comparisons between the re-determined SNPs’ causal structures was recorded as well, along with a mean relative structural Hamming distance (defined similarly as in the first pillar).

The reasoning underlying these metrics was to assess the impact inflicted on the similarity between SS0 and SS1 when k patients were randomly excluded from the corresponding datasets (n=267,n=597, respectively), thus providing an assessment of their structure’s similarity robustness.

(III) The third pillar of the analysis concerned the assessment of the robustness of the SNPs’ local causal structure around the discrete form of the Syntax Score, namely its Markov Blanket. The assessment of the Markov Blanket was performed only when Syntax Score was positive (n=597). In SS1, the Markov Blanket consisted of 4 SNPs, particularly, the “rs2046934”, “rs1803274”, “rs3184504”, and “rs1122608”. Then, a number of k=1,2,3,5,10,20,30,50 patients was randomly selected and excluded from the dataset, and the corresponding Markov Blanket of the Syntax Score was re-determined. The above procedure was repeated 100 times for each k. Then, for each k, several metrics were employed. Particularly, over the 100 iterations, the percentage of inclusion in the Markov Blanket of the Syntax Score for each of the four above-mentioned SNPs was computed, the percentage of inclusion in the Markov Blanket of exactly zero/one/two/three/four out of the four SNPs was recorded, and the mean number of SNPs other than the ones included in the initial Markov Blanket was computed as well.

**Figure 1:**
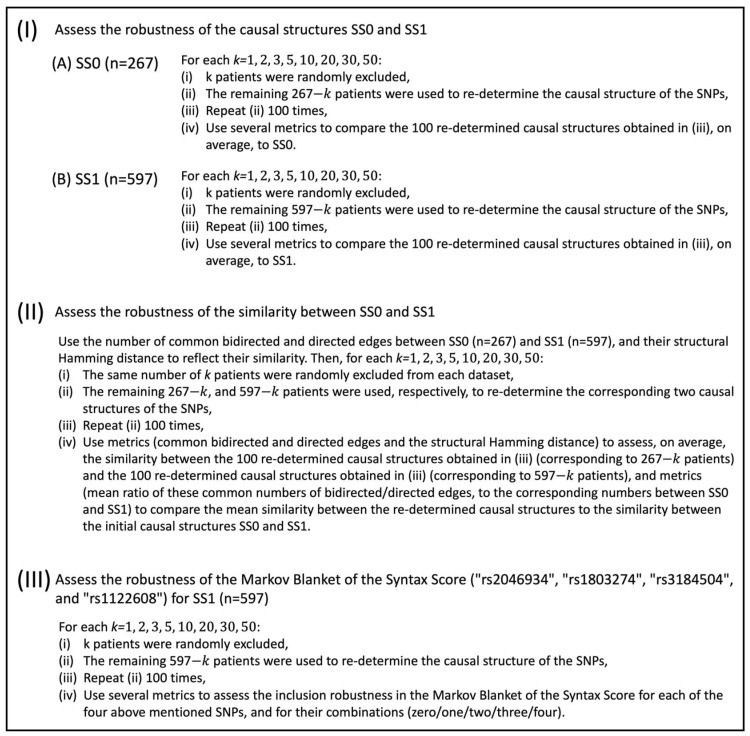
The schematic overview of the study flow.

The schematic overview of the study flow is displayed in Figure 1.

## 3. Results

The first pillar of the analysis concerned the assessment of the robustness of the SNPs’ initial causal structures, SS0 and SS1. The number of bidirected edges in the causal structures of the SNPs was 66 (SS0) and 72 (SS1). The corresponding numbers of directed edges were 40 (SS0) and 58 (SS1).

**Figure 2:**
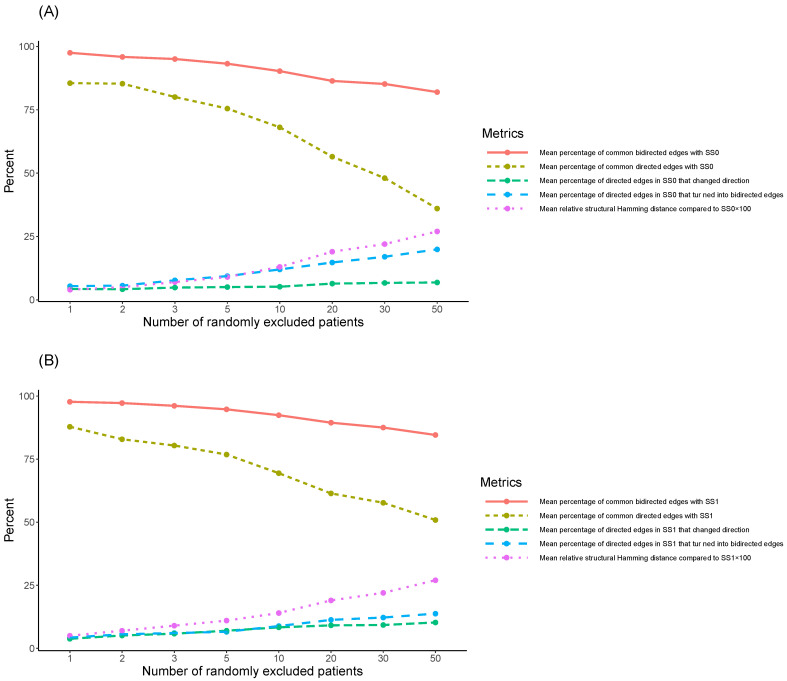
The metrics related to percentages are displayed that compare the initial causal structures of the SNPs, based on the datasets that correspond to Syntax Score being equal to zero (SS0—(**A**)) and one (SS1—(**B**)), with n=267 cases, and n=597 cases, respectively, to the mean behavior of the 100 new SNPs’ causal structures that correspond to the same datasets after randomly excluding k=1,2,3,5,10,20,30,50 patients in each case. Particularly, the mean percentage of bidirected edges that were in common in the 100 new SNPs’ causal structures with each initial causal structure; the mean percentage of directed edges that were in common in the 100 new SNPs’ causal structures with each initial causal structure; the mean percentage of directed edges in each initial causal structure that turned into bidirected edges in the 100 new SNPs’ causal structures; the mean percentage of directed edges in each initial causal structure that changed direction in the 100 new SNPs’ causal structures; the mean relative structural Hamming distance of the 100 new SNPs’ causal structures to the initial ones (i.e., the actual measured mean Hamming distance divided by the maximum value that it could potentially receive in each case, namely if all relations between nodes were altered in the 100 new SNPs’ causal structures compared to the initial ones). The mean relative structural Hamming distance was multiplied by 100 in the figure for visualization purposes. The number of bidirected edges in the initial causal structures of the SNPs was 66 (SS0—(**A**)) and 72 (SS1—(**B**)). The corresponding numbers of directed edges were 40 (SS0—(**A**)) and 58 (SS1—(**B**)).

As k was increasing, the causal structure of the SNPs was increasingly affected as well. Particularly, the mean number of bidirected/directed edges in the 100 new SNPs’ causal structures (corresponding to each k) that were in common with the initial causal structures, SS0 and SS1, respectively, exhibited a decreasing trend in both cases (see Table 1 and Figure 2). This decreasing trend was reflected as well in the corresponding mean percentages. On the other hand, the mean number of directed edges in both SSO and SS1 that turned into bidirected edges, or changed direction in the 100 new SNPs’ causal structures, and the corresponding mean percentages, exhibited an increasing trend (see Table 1).

More specifically, when considering SS0 (Table 1A and Figure 2A), the percentage of the common bidirected edges of the 100 new SNPs’ causal structures with SS0 exhibited its highest mean value at k=1 (97.48%), decreased for higher values of k, and exhibited its lowest mean value at k=50 (82.00%). On the other hand, the mean percentage of the common directed edges with SS0 exhibited a more intense decline over k, compared to the mean percentage of common bidirected edges with SS0, assuming its highest value at k=1 (85.53%), and its lowest value at k=50 (36.03%). The rather low mean value of the percentage of the common directed edges with SS0 at k=50 was further investigated and it was found that the mean percentage of directed edges in the initial causal structure, SS0, that turned into bidirected edges in the corresponding 100 new SNPs’ causal structures, was on average 19.90% at k=50 (5.40% at k=1). In addition, the mean percentage of directed edges in SS0 that changed direction in the 100 new SNPs’ causal structures was on average 6.85% at k=50 (4.30% at k=1). Thus, at k=50, a total mean percentage of 62.78% of the directed edges in the initial causal structure SS0 still corresponded to causal relationships in the respective 100 new SNPs’ causal structures. As shown in Figure 2A, the mean percentage of directed edges in SS0 that either turned into bidirected edges or changed direction in the corresponding 100 new SNPs’ causal structures exhibited an increasing trend over k. More specifically, at k=1,2,3,5,10,20,30,50, its corresponding values were 9.70%, 9.80%, 12.53%, 14.38%, 17.18%, 21.13%, 23.68%, and 26.75%. Thus, the mean percentage of the directed edges in the initial causal structure SS0 that still corresponded to causal relationships in the respective 100 new SNPs’ causal structures at k=1,2,3,5,10,20,30,50 was 95.23%, 95.10%, 92.58%, 89.86%, 85.26%, 77.63%, 71.71%, and 62.78%. Namely, the observed decreasing trend over k was much milder compared to the one related to the percentage of the common directed edges with SS0 over k, and similar to the one related to the percentage of the common bidirected edges with SS0 over k.

Interestingly, the increases/decreases in mean values in Figure 2A were all found to be monotonically increasing/decreasing over k (see also Table 1A). Both the mean percentage of common bidirected edges with SS0, and the summation of the mean percentages of the common directed edges with SS0, and of the directed ones in SS0 that either turned into bidirected edges or changed direction, exhibited a value over 85%, even at k=10.

In the case of SS1 (Table 1B and Figure 2B), the mean percentage of the common bidirected edges with SS1, across the corresponding (to each k) 100 new SNPs’ causal structures, exhibited its highest value at k=1 (97.72%), decreased for higher values of k, and exhibited its lowest value at k=50 (84.58%), similarly to the case of SS0. The mean percentage of the common directed edges with SS1 declined more drastically over k, and exhibited its highest value at k=1 (87.84%), decreased for higher values of k, and exhibited its lowest value at k=50 (50.88%). This percentage was higher than the corresponding one in the case of SS0 (36.03%). The mean percentage of directed edges in SS1 that turned into bidirected edges in the respective 100 new SNPs’ causal structures was on average 13.76% at k=50 (4.28% at k=1). Additionally, the mean percentage of directed edges in the initial causal structure, SS1, that changed direction in the 100 new SNPs’ causal structures was on average 10.26% at k=50 (3.79% at k=1). Thus, the total mean percentage of the directed edges in SS1 that still corresponded to causal relationships in the new SNPs’ causal structures at k=50 was 74.90%. As shown in Figure 2B, the mean percentage of directed edges in SS1 that either turned into bidirected edges or changed direction in the corresponding 100 new SNPs’ causal structures exhibited an increasing trend over k, that was similar to the trend of the corresponding mean percentage related to SS0. Particularly, at k=1,2,3,5,10,20,30,50, its corresponding values were 8.07%, 10.67%, 11.88%, 13.57%, 17.17%, 20.41%, 21.48%, and 24.02%. Consequently, the mean percentage of the directed edges in the initial causal structure SS1 that still corresponded to causal relationships in the respective 100 new SNPs’ causal structures at k=1,2,3,5,10,20,30,50 was 95.91%, 93.55%, 92.28%, 90.60%, 86.61%, 81.82%, 79.19%, and 74.90%. Namely, the observed decreasing trend over k was milder compared to the one related to the percentage of the common directed edges with SS1 over k, and similar to the one related to the percentage of the common bidirected edges with SS1 over k.

Similarly, as in the case of SS0, the increases/decreases in mean values were all monotonic over k (Figure 2B). Both the mean percentage of common bidirected edges with SS1, and the summation of the mean percentages of the common directed edges with SS1, and of the directed ones in SS1 that turned into bidirected edges or changed direction, exhibited a value over 85%, even at k=10 (similarly to in SS0).

All the above were reflected in the value of the mean structural Hamming distance of the 100 new SNPs’ causal structures compared to the initial ones, SS0 and SS1, and of the corresponding mean relative structural Hamming distance, which monotonically increased over k in both cases. In particular, the mean structural Hamming distance over the 100 new SNPs’ causal structures compared to SS0 increasingly assumed values from 10.46 at k=1 (mean relative value 0.04%), to 67.53 at k=50 (mean relative value 0.27%). In the case of SS1, it exhibited increasing values from 12.38 at k=1 (mean relative value 0.05%) to 67.84 at k=50 (mean relative value 0.27%).

**Figure A1:**
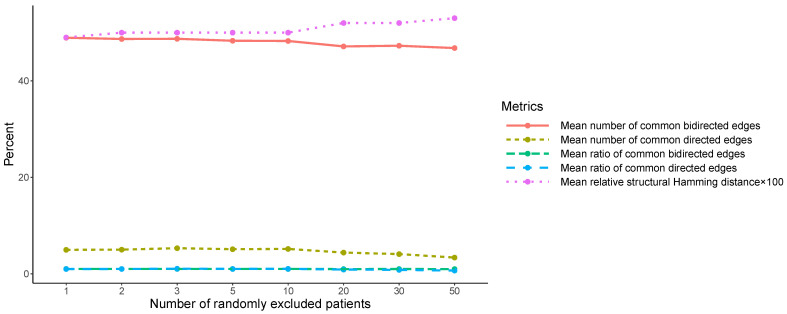
The metrics displayed assess the comparison between the causal structures of the SNPs that correspond initially to the datasets with Syntax Score being equal to zero (SS0) and one (SS1), with *n* = 267 cases, and *n* = 597 cases, respectively, after randomly excluding *k* = 1, 2, 3, 5, 10, 20, 30, 50 patients in each case. Particularly, the mean number of common bidirected and directed relationships, over the pairwise comparisons between the 100 new SNPs’ causal structures; the mean ratio of these common bidirected and directed relationships to the corresponding numbers of common bidirected and directed relationships, respectively, in the two initial SNPs’ causal structures; the mean structural Hamming distance over the pairwise comparisons between the 100 new SNPs’ causal structures corresponding to SS0 and SS1, and the corresponding mean relative structural Hamming distance (i.e., the actual measured mean Hamming distance divided by the maximum value that it could potentially receive, namely if all relations between nodes were altered between the compared SNPs’ causal structures). The mean relative structural Hamming distance was multiplied by 100 in the figure for visualization purposes. The numbers of common bidirected and directed edges in the comparison of the initial causal structures of the SNPs were 48 and 5.

The robustness of the similarity between the two initial SNPs’ causal structures, SS0 and SS1, was assessed within the second pillar of the analysis. SS0 and SS1 were compared, at first, using the structural Hamming distance, which was found to be 124. Additionally, the number of their common bidirected and directed relationships was computed (48 and 5, respectively). Then, the mean number of common bidirected and directed relationships, over the 100 pairwise comparisons (corresponding to each k) between the new SNPs’ causal structures, was found to be similar over k to the ones corresponding to SS0 and SS1 (48 and 5), and fluctuated slightly around these values (Table 2 and Figure A1). Particularly, the maximum observed mean number of common bidirected relationships was 48.94 (at k=1), and the minimum mean number was 46.80 (at k=50). Of all the values of the observed mean number at the different values of k, only the one at k=50 differed more than one from 48, which was the mean number of common bidirected edges between SS0 and SS1. Respectively, the maximum observed mean number of common directed edges was 5.31 (at k=3), and the minimum mean number was 3.37 (at k=50). Of all the values of the observed mean number of common directed edges, only the one at k=50 differed more than one in absolute value from 5, which corresponded to the mean number of common directed edges between SS0 and SS1. The corresponding mean ratios to 48 and 5, respectively (Table 2), were found to fluctuate in proximity to 1, with the exception of the mean ratio of the common number of directed edges at k=30,50, which exhibited a ratio value of 0.82 and 0.67, respectively. On the oher hand, the mean structural Hamming distance between the 100 re-determined SNPs’ causal structures exhibited a slight monotonic increase over k, from 125.05 at k=1 (mean relative value 0.49%) to 135.47 at k=50 (mean relative value 0.53%).

The assessment of the robustness of the SNPs’ Markov Blanket (rs2046934, rs1803274, rs3184504, and rs1122608) around the discrete form of the Syntax Score (when the Syntax Score was higher than 0) was performed within the third pillar of the analysis (Table 3). The rs2046934 was included in the Markov Blanket of the Syntax Score with a percentage of 100% at k=1,2,3,5, and was still included with a percentage of at least 90% until k=30 (Table 3). It exhibited its lowest percentage of inclusion at k=50, with 83%. Similarly, the rs1122608 was included in all 100 iterations in the Markov Blanket at k=1,2,3,5, and was included with a percentage of at least 90% until k=50 (Table 3). The rs1803274 and rs3184504 exhibited lower percentages of inclusion in the Markov Blanket compared to rs2046934 and rs1122608 (Table 3). Particularly, the maximum percentage for rs1803274 was 76% at k=1, and the minimum percentage was 50% at k=50. The maximum percentage for rs3184504 was 89% at k=1, and the minimum percentage was 42% at k=50. The rs3184504 exhibited higher inclusion percentages compared to rs1803274 at k=1,2,3,5,10,20. However, at k=30,50, its inclusion percentages in the Markov Blanket were lower compared to rs1803274. From the four SNPs constituting the initial Markov Blanket, rs1122608 exhibited the highest percentages of inclusion in the Markov Blanket over the different values of *k*.

Regarding the percentages of inclusion in the Markov Blanket of exactly zero/one/two/three/four out of the four SNPs (Table 3), it was found that the percentage none of the initial SNPs to be included in the Markov Blanket was zero at all values of k. Similarly, the percentage of only one of the initial SNPs to be included in the Markov Blanket was zero at all k, with the exception of k=50, when it was found to be equal to four. The percentage of exactly two of the initial SNPs to be included in the Markov Blanket exhibited an increasing trend over k, reaching 43.00% at k=50. Similarly, the percentage of exactly three of the initial SNPs to be included in the Markov Blanket exhibited an increasing trend over k and until k=20 (reaching 61.00%). However, at k=30,50, it exhibited a decrease, assuming values 54%, and 37%, respectively. The percentage wherein the Markov Blanket included all of the initial SNPs was 65% at k=1, gradually monotonically declined, and was equal to 16% at k=50. On the other hand, the mean number of SNPs in the Markov Blanket, other than the four included in the initial Markov Blanket, was found to be less than 0.03 until k=5, and received its maximum value, 0.62, at k=50.

## 4. Discussion

This study aimed to assess the robustness of the SNPs’ causal structures in patients with coronary artery disease. Along with 228 single nucleotide polymorphisms of 963 CAD patients from the GESS clinical trial, their corresponding value of the Syntax Score was taken into account. The robustness of the causal structure of the SNPs related to the Syntax Score was assessed within three different analysis pillars. At first, the causal structures, SS0 and SS1, were independently assessed. Then, the robustness of their similarity was evaluated. Third, the SNPs’ local causal structure around the discrete form of the Syntax Score (non-zero values) was assessed.

In the first pillar, it was found that, as the number of randomly excluded patients (from SS0, n=267, and SS1, n=597, respectively) increased, both initial causal structures, SS0 and SS1, were impacted. More specifically, as expected, the larger the k (stronger intervention), the larger the observed impact on the SNPs’ causal structure. By assessing the mean number of bidirected/directed edges (and the corresponding mean percentages), which were in common in the 100 newly developed SNPs’ causal structures (after excluding *k* patients at random) with the structures SS0 and SS1, respectively, a monotonic decreasing trend was observed in both cases (Table 1, Figure 2). This result was more intense in the case of the common number of directed edges. However, further investigation has shown that the latter was alleviated by the fact that, at the same time, the mean number of directed edges in the initial causal structures that changed direction or turned into bidirected edges was monotonically increasing, accounting for this more intense decreasing trend. In addition, the decrease in the common number of directed edges to the initial structures was more evident in the case of SS0. This may be due to the fewer number of cases/patients SS0 was built on, compared to SS1 (n=267 vs. n=597). Overall, it is shown that even for k=10, the impact on the common number of bidirected/directed relationships is limited, and it is more drastic for much larger values of k, such as 50.

Within the second pillar, the similarity between SS0 and SS1 was evaluated based on the mean number of common bidirected and directed relationships over k. The analysis has shown that neither the mean number of common bidirected/directed relationships, nor the corresponding mean ratios, are impacted, with the exception of the common directed edges at k=50.

In the third pillar, the interest was focused on the assessment of the local causal structure around the discrete Syntax Score, considering only Syntax Score values higher than zero. Particularly, the robustness of the Syntax Score’s Markov Blanket comprising 4 SNPs, i.e., “rs2046934”, “rs1803274”, “rs3184504”, and “rs1122608”, was assessed. It was found that the Markov Blanket was quite robust, even for large values of k. Interestingly, SNPs rs2046934 and rs1122608 were included in the Markov Blanket, in all 100 iterations, for k=1,2,3,5. The remaining SNPs, rs1803274 and rs3184504, exhibited lower percentages, but, still, their inclusion percentage was higher than approximately 70% over k=1,2,3,5, and they maintained an inclusion percentage of around 50%, even for k=50. At the same time, the percentage of the 4 SNPs being simultaneously included in the Syntax Score’s Markov Blanket was over 40% for all k=1,2,3,5.

It is shown that, overall, the causal structure of the SNPs, taking into account the Syntax Score of the CAD patients, although impacted when intervening in the size of the datasets by randomly excluding patients, still maintains a quite robust behavior. The impact was, as expected, more intense for very large values of k, such as 50, whereas for smaller values of k, it was much milder. Particularly, the local causal structure around the non-zero discrete Syntax Score was found to be robust, even for large values of k.

The main limitation of this study is that the values of k that represented the intervention level in the sensitivity analysis were arbitrarily set to be 1,2,3,5,10,20,30, and 50. However, despite the fact that the selected values of k were arbitrary, the main purpose was to investigate the local and global causal structure robustness, and the selected values of k appropriately represented a range from milder to stronger intervention.

By considering the biological and pharmacological significance of the four SNPs identified in our analysis, it must be emphasized that the single nucleotide polymorphism rs2046934 is related to the P2RY12 gene, which is expressed in the platelets and plays a crucial role in their aggregation. The latter biological process is implicated in the pathogenesis and progression of CAD. Moreover, the pharmacological effects of the drug clopidogrel which binds to and inhibits P2RY12 function are affected by this single nucleotide polymorphism in patients with acute coronary syndrome [26,27]. The single nucleotide polymorphism rs1122608 is associated with the gene locus of low-density lipoprotein receptors and has been correlated with patients’ blood lipid profiles as well as CAD incidence [28]. Additionally, the single nucleotide polymorphism rs3184504 has been shown to be related to individuals with risk of CAD, as well as to increased blood LDL and diastolic blood pressure [29]. Last, the single nucleotide polymorphism rs1803274 is implicated with an increased risk of coronary in-stent restenosis [30]. As a matter of fact, the four SNPs, i.e., “rs2046934”, “rs1803274”, “rs3184504”, and “rs1122608”, might have an effect on the Syntax Score by affecting biological processes, such as blood thrombosis and dysfunction of lipid metabolism, that contribute to atheromatosis and thus lead to CAD risk, severity, and progression [31].

The work presented here permits the validation of important, previously identified, genetic risk factors affecting the Syntax Score, in a way that can facilitate the development of accurate risk-stratification algorithms applicable to the clinical setting. Especially, the results obtained contribute to the advancement of data analytics and algorithm development with the aim of extracting significant molecular knowledge relevant to CAD patient handling and therapeutics decisions to predict severity and complexity of the disease. Additionally, through this work, the advancement of machine learning (ML) feature selection algorithms and predictive modeling can be beneficial, by validating the optimal set of risk factors capable to predict future cardiovascular events in individuals and specific populations. Such an attempt has been recently presented with the ML model CRISSPAC (Coronary artery disease Risk-stratification Syntax Score Predictive Algorithm Calculator), developed to facilitate practitioners’ predicting the Syntax Score and severity of CAD [32]. Moreover, clinicians can apply these risk-stratification models at the point-of-care to explore disease heterogeneity seen within the clinical phenotypes, and thus improve therapy outcomes.

## 5. Conclusions

Causal discovery is a powerful tool with wide potential in the field of biology. The causal structure in a real-life dataset may validate expectations and/or provide insights into hidden knowledge, aiding, in different layers, scientific interpretations and conclusions. The robustness of the causal structure of the SNPs along with the Syntax Score of CAD patients was assessed herein, and it was found to be quite satisfactory within different schemes, particularly under milder interventions. Consequently, causal models may be used in this context, leveraging the understanding of biological aspects of coronary artery disease.

## Figures and Tables

**Table 1:** The metrics are displayed that assessed the comparison of the initial causal structures of the SNPs, based on the datasets that correspond to Syntax Score being equal to zero (SS0) and one (SS1), with n=267 cases, and n=597 cases, respectively, to the mean behavior of the 100 new SNPs’ causal structures that correspond to the same datasets after randomly excluding k=1,2,3,5,10,20,30,50 patients in each case. These metrics were computed independently for SS0 and SS1. Particularly, the mean number of bidirected edges that were in common in the 100 new SNPs’ causal structures with each initial causal structure, and the corresponding mean percentage; the mean number of directed edges that were in common in the 100 new SNPs’ causal structures with each initial causal structure, and the corresponding mean percentage; the mean number of directed edges in each initial causal structure that turned into bidirected edges in the 100 new SNPs’ causal structures, and the corresponding mean percentage; the mean number of directed edges in each initial causal structure that changed direction in the 100 new SNPs’ causal structures, and the corresponding mean percentage; the mean structural Hamming distance of the 100 new SNPs’ causal structures compared to each initial one, and the corresponding mean relative structural Hamming distance (i.e., the actual measured mean Hamming distance divided by the maximum value that it could potentially receive in each case, namely if all relations between nodes were altered in the 100 new SNPs’ causal structures compared to the initial ones). The number of bidirected edges in the initial causal structures of the SNPs was 66 (SS0) and 72 (SS1). The corresponding numbers of directed edges were 40 (SS0) and 58 (SS1).

(A) Assessment of the SNPs’ Causal Structure in Case the Syntax Score Was Zero (*n* = 267 Cases)–SS0								
k	1	2	3	5	10	20	30	50
Mean number of common bidirected edges with SS0	64.34	63.28	62.72	61.5	59.57	57.02	56.23	54.12
Mean percentage of common bidirected edges with SS0 (%)	97.48	95.88	95.03	93.18	90.26	86.39	85.20	82.00
Mean number of common directed edges with SS0	34.21	34.12	32.02	30.19	27.23	22.60	19.21	14.41
Mean percentage of common directed edges with SS0 (%)	85.53	85.30	80.05	75.48	68.08	56.50	48.03	36.03
Mean number of directed edges in SS0 that turned into bidirected edges	2.16	2.24	3.08	3.74	4.80	5.89	6.80	7.96
Mean percentage of directed edges in SS0 that turned into bidirected edges (%)	5.40	5.60	7.70	9.35	12.00	14.73	17.00	19.90
Mean number of directed edges in SS0 that changed direction	1.72	1.68	1.93	2.01	2.07	2.56	2.67	2.74
Mean percentage of directed edges in SS0 that changed direction (%)	4.30	4.20	4.83	5.03	5.18	6.40	6.68	6.85
Mean structural Hamming distance compared to SS0	10.46	13.69	17.95	23.58	32.87	47.05	54.56	67.53
Mean relative structural Hamming distance compared to SS0 (%)	0.04	0.05	0.07	0.09	0.13	0.19	0.22	0.27
**(B) Assessment of the SNPs’ Causal Structure in Case the Syntax Score Was Positive (*n* = 597 Cases)–SS1**								
k	**1**	**2**	**3**	**5**	**10**	**20**	**30**	**50**
Mean number of common bidirected edges with SS1	70.36	70.00	69.22	68.21	66.54	64.41	63.01	60.90
Mean percentage of common bidirected edges with SS1 (%)	97.72	97.22	96.14	94.74	92.42	89.46	87.51	84.58
Mean number of common directed edges with SS1	50.95	48.07	46.63	44.56	40.27	35.62	33.47	29.51
Mean percentage of common directed edges with SS1 (%)	87.84	82.88	80.40	76.83	69.43	61.41	57.71	50.88
Mean number of directed edges in SS1 that turned into bidirected edges	2.48	3.23	3.52	3.81	5.13	6.55	7.09	7.98
Mean percentage of directed edges in SS1 that turned into bidirected edges (%)	4.28	5.57	6.07	6.57	8.84	11.29	12.22	13.76
Mean number of directed edges in SS1 that changed direction	2.20	2.96	3.37	4.06	4.83	5.29	5.37	5.95
Mean percentage of directed edges in SS1 that changed direction (%)	3.79	5.10	5.81	7.00	8.33	9.12	9.26	10.26
Mean structural Hamming distance compared to SS1	12.38	17.62	21.79	26.75	36.55	49.23	55.77	67.84
Mean relative structural Hamming distance compared to SS1 (%)	0.05	0.07	0.09	0.11	0.14	0.19	0.22	0.27

**Table 2:** The metrics are displayed that assessed the similarity between the causal structures that initially corresponded to the datasets with Syntax Score being equal to zero (SS0) and positive (SS1), with n=267 cases and n=597 cases. After randomly excluding k=1,2,3,5,10,20,30,50 patients at the same time from each dataset, the similarity, for each k, was assessed based on the 100 pairwise comparisons, between the re-determined SNPs’ causal structures corresponding to SS0 and SS1. Particularly, the mean number of common bidirected and directed relationships, over the 100 pairwise comparisons, between the new SNPs’ causal structures corresponding to SS0 and SS1; the mean ratio of these common numbers of bidirected and directed relationships to the corresponding numbers of common bidirected and directed relationships, respectively, between SS0 and SS1; the mean structural Hamming distance over the 100 pairwise comparisons, between the new SNPs’ causal structures corresponding to SS0 and SS1, and the corresponding mean relative structural Hamming distance (i.e., the actual measured mean Hamming distance divided by the maximum value that it could potentially receive, namely if all relations between nodes were altered between the compared SNPs’ causal structures). The numbers of common bidirected and directed edges between SS0 and SS1 were 48 and 5, respectively.

Assessment of the Similarity between the Causal Structures of the SNPs in Case the Syntax Score Was Equal to Zero (*n* = 267 Cases), and Positive (*n* = 597 Cases)								
k	1	2	3	5	10	20	30	50
Mean number of common bidirected edges	48.94	48.68	48.72	48.32	48.27	47.14	47.28	46.80
Mean ratio of the common number of bidirected edges to the common number of bidirected edges between SS0 and SS1	1.02	1.01	1.02	1.01	1.01	0.98	0.99	0.98
Mean number of common directed edges	4.97	5.01	5.31	5.10	5.16	4.40	4.09	3.37
Mean ratio of the common number of directed edges to the common number of directed edges between SS0 and SS1	0.99	1.00	1.06	1.02	1.03	0.88	0.82	0.67
Mean structural Hamming distance	125.05	127.06	127.04	127.23	127.86	131.70	132.15	135.47
Mean relative structural Hamming distance (%)	0.49	0.50	0.50	0.50	0.50	0.52	0.52	0.53

**Table 3:** Several metrics are displayed that assess the robustness of the SNPs’ Markov Blanket around the discrete form of the Syntax Score, for the case that Syntax Score was higher than 0. The initial Markov Blanket (n=597 patients) consisted of 4 SNPs, particularly the “rs2046934”, “rs1803274”, “rs3184504”, and “rs1122608”. After randomly excluding k=1,2,3,5,10,20,30,50 patients, the corresponding Markov Blanket of the Syntax Score was re-determined (procedure was repeated 100 times for each *k*). For each *k*, the percentage of inclusion in the Markov Blanket of the Syntax Score for each of the four above-mentioned SNPs, the percentage of inclusion in the Markov Blanket of exactly zero/one/two/three/four out of the four SNPs, and the mean number of SNPs other than the ones included in the initial Markov Blanket are displayed.

Assessment of the Syntax Score’s Markov Blanket within SS1 (*n* = 597 Cases)								
k	1	2	3	5	10	20	30	50
rs2046934 (%)	100	100	100	100	96	97	90	83
rs1803274 (%)	76	70	69	68	59	59	52	50
rs3184504 (%)	89	85	74	69	62	60	48	42
rs1122608 (%)	100	100	100	100	98	97	96	90
0 out of 4 (%)	0	0	0	0	0	0	0	0
1 out of 4 (%)	0	0	0	0	0	0	0	4
2 out of 4 (%)	0	0	0	5	16	13	30	43
3 out of 4 (%)	35	45	57	53	53	61	54	37
4 out of 4 (%)	65	55	43	42	31	26	16	16
Mean number of SNPs other than the ones included in the initial Markov Blanket	0.02	0.01	0.01	0.03	0.13	0.20	0.35	0.62

## Data Availability

Data available on request from the corresponding author.

## References

[1] Arup K.M., Debashree C., Binata H., Prosenjit P., Arif U., Supriyo C. (2019). A review on coronary artery disease, its risk factors, and therapeutics. J. Cell. Physiol..

[2] Amit K.V., Sekar K. (2017). Genetics of coronary artery disease: Discovery, biology and clinical translation. Nat. Rev. Genet..

[3] Kessler T., Schunkert H. (2021). Coronary Artery Disease Genetics Enlightened by Genome-Wide Association Studies. J. Am. Coll Cardiol. Basic Trans. Sci..

[4] Chatzopoulou F., Kyritsis K.A., Papagiannopoulos C.I., Galatou E., Mittas N., Theodoroula N.F., Papazoglou A.S., Karagiannidis E., Chatzidimitriou M., Papa A. (2022). Dissecting miRNA–Gene Networks to Map Clinical Utility Roads of Pharmacogenomics-Guided Therapeutic Decisions in Cardiovascular Precision Medicine. Cells.

[5] Ganopoulou M., Chatzopoulou F., Mittas N., Giannopoulos-Dimitriou A., Saiti A., Papazoglou A.S., Karagiannidis E., Chatzidimitriou D., Gounaris A., Sianos G. Exploration of causal relations between Genomic Biomarkers and the Syntax Score in Cardiovascular Diseases.

[6] Vizirianakis I.S., Chatzopoulou F., Papazoglou A., Karagiannidis E., Sofidis G., Stalikas N., Stefopoulos C., Kyritsis K., Mittas N., Theodoroula N. (2021). The GEnetic Syntax Score: A genetic risk assessment implementation tool grading the complexity of coronary artery disease-rationale and design of the GESS study. BMC Cardiovasc. Disord..

[7] Chatzopoulou F., Mittas N., Giannopoulos-Dimitriou A., Saiti A., Ganopoulou M., Karagiannidis E., Papazoglou A.S., Stalikas N., Papa A., Chatzidimitriou D. Improving Syntax Score Prediction by Integrating Genomic Biomarkers Data into Machine Learning Risk-stratification Models of Practical Utility to Precision Cardiovascular Medicine. submitted.

[8] Li J., Liu L., Le T.D. (2015). Practical Approaches to Causal Relationship Exploration.

[9] Ganopoulou M., Michailidis M., Angelis L., Ganopoulos I., Molassiotis A., Xanthopoulou A., Moysiadis T. (2021). Could Causal Discovery in Proteogenomics Assist in Understanding Gene–Protein Relations? A Perennial Fruit Tree Case Study Using Sweet Cherry as a Model. Cells.

[10] Skodra C., Michailidis M., Moysiadis T., Stamatakis G., Ganopoulou M., Adamakis I., Angelis E., Ganopoulos I., Tanou G., Samiotaki M. (2022). Disclosing the molecular basis of salinity priming in olive trees using proteogenomic model discovery. Plant Physiol..

[11] Boutsika A., Michailidis M., Ganopoulou M., Dalakouras A., Skodra C., Xanthopoulou A., Stamatakis G., Samiotaki M., Tanou G., Moysiadis T. (2023). A wide foodomics approach coupled with metagenomics elucidates the enviromental signature of potatoes. iScience.

[12] Piccininni M., Konigorski S., Rohmann J., Kurth T. (2020). Directed acyclic graphs and causal thinking in clinical risk prediction modeling. BMC Med. Res. Methodol..

[13] Raghu V.K., Zhao W., Pu J., Leader J., Wang R., Herman J., Yuan J., Benos P., Wilson D. (2019). Feasibility of lung cancer prediction from low-dose CT scan and smoking factors using causal models. Thorax.

[14] Ganopoulou M., Kagkelidis I., Sianos G., Angelis L. (2021). Causal Models for the Result of Percutaneous Coronary Intervention in Coronary Chronic Total Occlusions. Appl. Sci..

[15] Pellet J.P., Elisseeff A. (2008). Using Markov Blankets for Causal Structure Learning. J. Mach. Learn. Res..

[16] Aliferis C.F. (2010). Local causal and Markov blanket induction for causal discovery and feature selection for classification part II: Analysis and extensions. J. Mach. Learn. Res..

[17] Ling Z., Yu K., Zhang Y., Liu L., Li J. (2022). Causal learner: A toolbox for causal structure and markov blanket learning. Pattern Recognit. Lett..

[18] Wang H., Ling Z., Yu K., Wu X. (2020). Towards efficient and effective discovery of Markov blankets for feature selection. Inf. Sci..

[19] Gao T., Ji Q. (2017). Efficient score-based Markov Blanket discovery. Int. J. Approx. Reason..

[20] Pearl J. (2009). Causality.

[21] Neopolitan R.E. (2003). Learning Bayesian Networks.

[22] Spirtes P., Glymour C.C., Scheines R. (2000). Causation, Prediction, and Search.

[23] Kalisch M., Hauser A., Maathuis M., Machler M. (2020). An Overview of the Pcalg Package for R. https://cran.r-project.org/web/packages/pcalg/vignettes/vignette2018.pdf.

[24] Tsagris M., Bordoudakis G., Lagani V., Tsamardinos I. (2018). Constraint-based causal discovery with mixed data. Int. J. Data Sci. Anal..

[25] Tsamardinos I., Brown L.E., Aliferis C.F. (2006). The max-min hill-climbing Bayesian network structure learning algorithm. Mach. Learn..

[26] Cavallari U., Trabetti E., Malerba G., Biscuola M., Girelli D., Olivieri O., Martinelli N., Angiolillo D., Corrocher R., Pignatti P. (2007). Gene sequence variations of the platelet P2Y12 receptor are associated with coronary artery disease. BMC Med. Genet..

[27] Cuisset T., Frere C., Quilici J., Morange P., Saut N., Lambert M., Camoin L., Vague I., Bonnet J., Alessi M. (2007). Role of the T744C polymorfism of the P2Y12 gene on the platelet response to a 600-mg loanding dose of clopidogrel in 597 patients with non-ST-segment elevation acute coronary syndrome. Thromb. Res..

[28] Liu S., Xiu B., Liu J., Xue A., Tang Q., Shen Y., Xie J. (2016). Association of rs1122608 with Coronary Artery Disease and Lipid Profile: A Meta-analysis. Arch. Med. Res..

[29] Aghabozorg A., Ghaderian S., Mirfakhraie R., Piryaei M., Zaim Kohan H. (2014). Association Study of rs3184504 C>T Polymorphism in Patients with Coronary Artery Disease. Int. J. Mol. Cell Med..

[30] Leva L., Kovarova P., Faldynova L., Plevova P., Hilscherova S., Zapletalova J., Kusnierova P., Kukla P. (2015). The rs1803274 polymorphism of the BCHE gene is associated with an increased risk of coronary in-stent restenosis. BMC Cardiovasc. Disord..

[31] Mittas N., Chatzopoulou F., Kyritsis K.A., Papagiannopoulos C.I., Theodoroula N.F., Papazoglou A.S., Karagiannidis E., Sofidis G., Moysidis D.V., Stalikas N. (2022). A Risk-stratification machine learning framework for the prediction of coronary artery disease severity: Insights from the GESS trial. Front. Cardiovasc. Med..

[32] Mittas N., Chatzopoulou F., Karagiannidis E., Chatzidimitriou D., Sianos G., Angelis L., Vizirianakis I.S. (2023). CRISSPAC: A web-based platform for predicting the SYNTAX Score and severity of coronary artery disease. SoftwareX.

